# Comparative Analysis of Bone Marrow, cfDNA and CTCs for NGS-Based Multiple Myeloma Detection: A Pilot Study Indicating the Potential of CTCs

**DOI:** 10.3390/cancers17244008

**Published:** 2025-12-16

**Authors:** Wouter De Brouwer, Robbe Heestermans, Jona Van der Straeten, Kiara Falise, Ann De Becker, Isabelle Vande Broek, Rik Schots, Marleen Bakkus, Ivan Van Riet

**Affiliations:** 1 Research Group Hematology-Immunology, Universitair Ziekenhuis Brussel (UZ Brussel), Vrije Universiteit Brussel (VUB), Laarbeeklaan 101, 1090 Brussels, Belgium; wouter.debrouwer@uzbrussel.be (W.D.B.); marleen.bakkus@uzbrussel.be (M.B.); 2 Department of Hematology, Universitair Ziekenhuis Brussel (UZ Brussel), Vrije Universiteit Brussel (VUB), Laarbeeklaan 101, 1090 Brussels, Belgium; 3 Department of Clinical Biology, Universitair Ziekenhuis Brussel (UZ Brussel), Vrije Universiteit Brussel (VUB), Laarbeeklaan 101, 1090 Brussels, Belgium; 4 Department of Hematology, VITAZ, Moerlandstraat 1, 9100 Sint-Niklaas, Belgium

**Keywords:** multiple myeloma, liquid biopsy, circulating tumor cells, cell free DNA, minimal residual disease

## Abstract

**Background/Objectives**: Minimal residual disease (MRD) persists in most multiple myeloma (MM) patients, causing relapse despite deep remissions. Repeatability of MRD detection in MM bone marrow (BM) samples is limited, underscoring the need for blood-based monitoring approaches that can allow more thorough disease surveillance. **Methods**: This study compares tumor detection rates in BM-derived DNA with different blood-derived DNA sources, using next-generation sequencing of the immunoglobulin locus (NGS-IG). CD138-targeted immunomagnetic enrichment of circulating tumor cells (CTCs) followed by vacuum evaporation to concentrate DNA was used to optimize the tumor detection rate. **Results**: Tumor DNA was detected in 76%, 88% and 100% of cell-free DNA, peripheral blood-derived mononuclear cells, DNA and enriched CTC-DNA samples of patients with active myeloma, respectively. These data indicate that enriched CTC samples were the most informative for evaluation of disease detection with NGS-IG in patients with active myeloma. MRD detection was performed in paired BM and enriched CTC-DNA samples from 37 patients in remission. MRD positivity was found in the BM of 24 patients, with half of them (12/24) also showing the presence of MRD when enriched CTC-DNA was used. Interestingly, time to progression (TTP) of enriched CTC MRD-negative/BM MRD-positive patients was comparable to that of double MRD-negative (BM and CTC) patients. Moreover, double positive patients showed a trend to earlier relapses. **Conclusions**: Our data suggest that NGS-IG analysis with enriched CTC-DNA may offer improved predictive abilities for relapse in multiple myeloma compared to the currently used BM-DNA-based tumor detection method.

## Simple Summary

Despite effective therapies, multiple myeloma remains an incurable disease. Even when achieving deep remissions, almost all patients eventually relapse. Current evaluation of this persistent disease is based on bone marrow evaluation, but this approach has significant drawbacks. Blood-based disease evaluation is less invasive and potentially more comprehensive in evaluation of total tumor mass but lacks sensitivity. It is currently unknown which biomarker in blood is superior. Therefore, we tested three different blood-derived DNA sources: peripheral blood mononuclear cells, enriched circulating tumor cells (CTCs) and cell-free DNA. Enrichment of CTCs, followed by next-generation sequencing, resulted in the highest sensitivity. In patients with detectable disease in their bone marrow, but no detectable CTCs, the first relapse occurred after almost 4 years, while early relapse (<18 months) occurred in 5/12 patients with detectable CTCs.

## 1. Introduction

Multiple myeloma (MM) is characterized by an accumulation of monoclonal plasma cells in the bone marrow (BM) which produce a monoclonal protein, measurable in peripheral blood [1,2]. This disease remains incurable despite the increasing effectiveness of therapy [3]. Widespread, multifocal disease (both within and outside of the BM) and novel, more efficient therapies, pose challenges to both conventional and recent methods for response assessment. Conventional response criteria based on indirect disease evaluation (by measurement of urinary or plasma monoclonal protein) lack sensitivity in the current treatment era. Therefore, more sensitive evaluation techniques such as minimal residual disease (MRD) assessment on BM samples have been developed. Next-generation sequencing (NGS) of the individual immunoglobulin genes (*IG*) has been one of the most sensitive MRD detection methods to date, capable of detecting one tumor cell in 10^5^–10^6^ normal cells [4]. Despite its sensitivity and specificity, BM MRD analysis is limited in its evaluation of the disease. The use of single-site BM samples does not capture spatial heterogeneity, which is frequently observed in MM [5,6]. Moreover, extramedullary plasmacytomas, found in about 3% of patients at diagnosis and increasingly frequent as disease progresses, remain undetected with this technique [7]. Collection of BM aspirates is an invasive procedure requiring skilled personnel and has limited repeatability. Furthermore, this method is susceptible to pre-analytical errors, such as hemodilution, which can reduce sensitivity [5]. As a result, liquid biopsies are being investigated as an alternative for tumor detection in myeloma BM samples, both during active disease and remission.

Liquid biopsies allow us to obtain tumor-derived material for pathological evaluation from various bodily fluids [8,9]. Studied matrices include cell-free DNA (cfDNA), circulating tumor cells (CTCs), various circulating RNA subtypes and extracellular vesicles. cfDNA has already been accepted as an alternative to tissue biopsy for mutation detection in several solid tumors [10,11,12,13]. Due to the high specificity of the rearranged *IG* gene, both cfDNA and CTCs-derived DNA are particularly promising matrices for future liquid biopsy studies in MM. Due to the anatomic proximity of BM and the vascular compartment, liquid biopsies seem even more promising in MM, as suggested by higher cfDNA concentrations compared to solid tumors as well as the presence of CTCs in virtually all MM patients [14,15,16,17,18,19]. Initially, myeloma cells are strongly dependent on the bone marrow, which provides growth signals and resistance to chemotherapy [20,21,22]. However, as the disease progresses, malignant plasma cells can egress out of the BM, circulate and invade distant sites [16,21]. At diagnosis, CTCs can be detected in almost all MM patients and higher CTCs numbers are associated with a worse prognosis [16,23,24,25,26,27]. Through hematogenous dissemination, CTCs are believed to give rise to extramedullary plasmacytomas, which remain undetected with current BM MRD strategies [26]. Recently, several studies have evaluated the clinical value of different liquid biopsy-derived biomarkers for disease monitoring in MM, but thorough comparative studies are very limited and show conflicting observations [19,28,29,30,31,32,33,34,35,36].

In this article, we compare cellular DNA derived from peripheral blood (PB) and immunomagnetically enriched circulating tumor cells with cfDNA as potential biomarkers to detect clonally rearranged immunoglobulin gene sequences found in the BM of MM patients.

## 2. Materials and Methods

### 2.1. Spike Experiments

Peripheral blood mononuclear cells (PBMNCs) of normal donors were isolated by density gradient centrifugation (Lymphoprep, STEMCELL technologies, Cologne, Germany). Next, cultured MM cells (LP-1 cell line) were added to 100 million PBMNCs in various concentrations (10^−2^ to 10^−7^) [37]. A small fraction was not enriched, but most cells were immunomagnetically labeled with CD138 Microbeads according to the manufacturer’s protocol (Miltenyi Biotec, Leiden, The Netherlands). Briefly, cells were washed and resuspended in running buffer (Miltenyi), with the addition of a blocking reagent (Miltenyi) and CD138 Microbeads (volumes were scaled to cell numbers). Cells were incubated for 15 min, washed and resuspended in 500 µL of running buffer prior to immunomagnetic separation (“Posseld”) by the AutoMacs device (Miltenyi). CD138 positive cells were isolated, using the “Posseld” program on the AutoMacs device (Miltenyi), according to the manufacturer’s protocol. Cells were cytospun onto slides for microscopic evaluation of purity and yield before and after enrichment. For the mixtures with 10^−5^ to 10^−7^ LP-1 cells, the elution volume was reduced and supplemented with 200 µL of AL buffer (Qiagen, Venlo, The Netherlands) prior to DNA extraction and evaluation with qASO-PCR.

### 2.2. Collection and Processing of Patient Samples

The study protocol was approved by the ethical committees of Universitair Ziekenhuis Brussel (Brussels) and AZ Nikolaas (Sint-Niklaas) (B.U.N. 14321733078). All patients signed the informed consent form prior to sampling. Paired BM aspirate and PB samples were compared for 33 patients. Additionally, remission samples from 37 patients (17 very good partial response, 20 in (s)CR) were collected. PB from six healthy volunteers was used as negative control and/or to evaluate vacuum evaporation.

**Figure 1:**
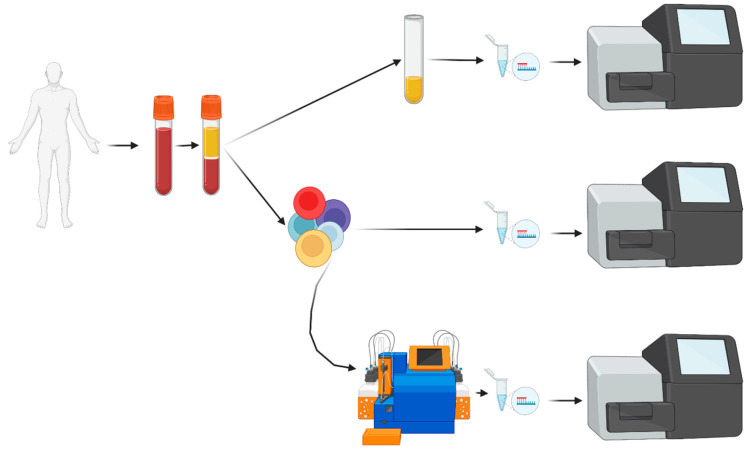
Flowchart of liquid biopsy (PB) sample processing. After obtaining informed consent, blood was collected and, after centrifugation, plasma was separated. Cell-free DNA was extracted and analyzed by NGS. From the cellular fraction, mononuclear cells were enriched with Ficoll density centrifugation. A small fraction of PBMNCs was processed for direct DNA extraction and NGS analysis. All other PBMNCs were stained with immunomagnetic beads, separated over the AutoMacs Pro device followed by DNA extraction, evaporation of excess free water and NGS analysis. PBMNCs: peripheral blood mononuclear cells; NGS: next-generation sequencing.

Bone marrow and PB were collected in EDTA (S-Monovette EDTA, Sarstedt, Nümbrecht, Germany). Higher volumes of PB were collected for patients in remission (100 mL) vs. NDMM/RRMM patients (25 mL). All samples were processed within 24 h. An overview of all sample processing steps is presented in Figure 1. PB was centrifuged, separated plasma was centrifuged twice more—low speed (2095 *g*) and high speed (13,200 *g*)—for 10 min at 4 °C. Samples were stored at −20 °C. Mononuclear cells from PB and BM were separated by density gradient centrifugation. Up to five million PBMNCs were separated and stored in 200 µL of PBS and 200 µL of Lysis Buffer (Qiagen) at −20 °C. Residual cells were labeled, separated and stored as described previously. BM MNCs were stored in 100 µL of PBS (Gibco, Bleiswijk, The Netherlands) and 900 µL of RNAlater (Life Technologies, Bleiswijk, The Netherlands) at −20 °C.

### 2.3. Extraction of Cellular and Cell-Free DNA

Cellular DNA, was extracted with the QIAamp DNA mini kit (Qiagen, Venlo, The Netherlands). DNA concentrations were determined by Nanophotometric analysis (NanoPhotometer Pearl^®^, Implen, Munich, Germany). cfDNA was extracted with the QIAamp Circulating Nucleic Acid Kit (Qiagen). DNA concentration was quantified by the Qubit dsDNA HS Assay Kit (ThermoFisher Scientific, Merelbeke, Belgium).

### 2.4. Vacuum Evaporation

PBMNC-DNA from 4 healthy donors was eluted in 200 µL of AE buffer (Qiagen) or 200 µL of DNAse- and RNAse-free water (DNAse FW, MP Biomedicals Europe, Eschwege, Germany). Next, DNA was concentrated 4.5 times with the RVC 2-25 CDplus vacuum concentrator (Martin Christ, Osterode am Harz, Germany). The DNA concentration was checked with albumin PCR before and after evaporation. For patients’ enriched CTC samples, elution volume was reduced 3.3-fold.

### 2.5. Quantitative Allele Specific Oligonucleotide PCR (qASO-PCR)

qASO-PCR of the rearranged *IG*-region of the LP-1 cell line was performed as previously described [38]. Briefly, clonal *IG* rearrangements were determined by NGS and annotated by the IMGT/V-QUEST information system^®^. The primer design was carried out with the Oligo 7 primer analysis software version 7.60 (Molecular Biology Insights, Colorado Springs, CO, USA). A standard curve was made, all samples were run in triplicate and every reaction contained 600 ng DNA—quantified by the NanoPhotometer Pearl^®^. Results were interpreted according to the EuroMRD-ALL guidelines [39].

### 2.6. Immunoglobulin-Targeted Next-Generation Sequencing

Our in-house NGS method had a higher applicability for myeloma patients compared to qASO-PCR [38]. Therefore, all patient samples were examined with this *IG*-targeted NGS-method. Framework 3 (Fr3) primers were added, resulting in evaluation of the *IGH* and *IGK* genes by the LymphoTrack Dx *IGH* Framework 1 (Fr1)/Framework 3 (Fr3)/*IGK* Assay Panel MiSeq kits (Invivoscribe, San Diego, CA, USA). *IGH* (Fr1 and Fr3) and *IGK* rearrangements were identified with NGS in the NDMM and RRMM BM samples. A 25 µL PCR reaction containing up to 50 ng of DNA was performed and a minimum of 20.000 reads were aimed for. A prior diagnostic sample was used for patients in remission. Sequences which constituted ≥5.00% of total reads were defined as clonal sequences [40]. Next, for patients with active disease, the index clones were tracked in three paired liquid biopsies (Fr1, Fr3 and/or IGK depending on clonotype). The NGS-PCR total reaction volume was 50 µL, and reference molecules (gBlocks) were added as an internal control and calibrator. For all “active disease” samples, at least 100.000 reads were aimed for. All remission samples (BM and liquid biopsies) were analyzed in duplicate (with gBlocks) and at least 250.000 reads were aimed for. Samples were considered MRD-positive if at least 2 sequences, identical to the clonotype (identified in the diagnostic bone marrow sample) were detected.

### 2.7. Statistical Analysis

The McNemar test was used to compare paired binomial data. Paired and independent quantitative data were tested with the Wilcoxon signed-rank test and Mann–Whitney U test. A Chi-square was used for comparison of DNA levels from different sources. Kaplan–Meier analysis was used for survival analysis; significance was calculated with the log-rank test and Gehan–Breslow–Wilcoxon test. All statistical analysis were performed with IBM SPSS Statistics (version 27.0.1.0).

## 3. Results

### 3.1. Development of Dual Platform Strategy to Isolate DNA from Circulating Tumor Cells (CTCs)

#### 3.1.1. Immunomagnetic CTCs Enrichment with Immunomagnetic Beads

**Figure 2:**
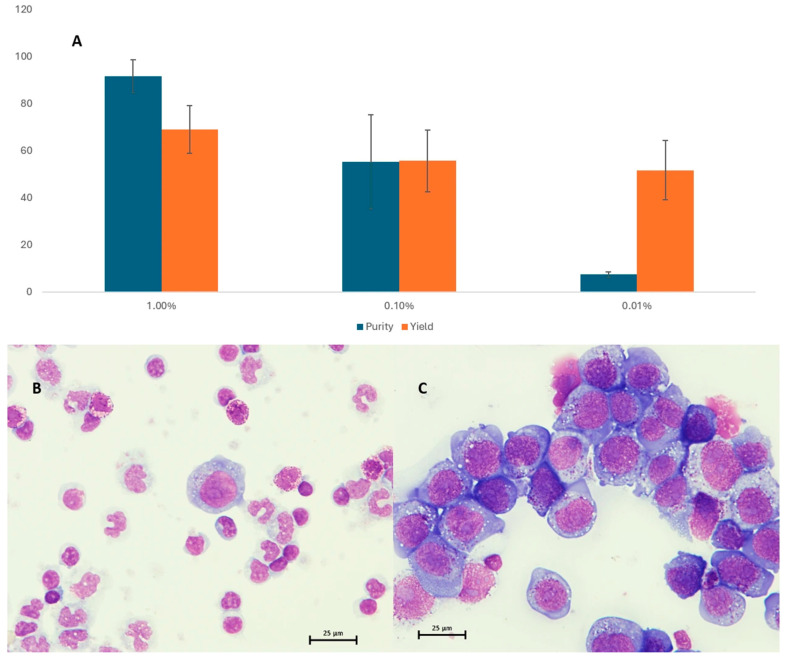
Purity and yield as assessed by the light microscopy are shown in panel (**A**). Panel (**B**,**C**) give a representative image of spiked mixtures before and after immunomagnetic enrichment, respectively.

A series of spike-in experiments with LP-1 myeloma cells evaluated immunomagnetic enrichment of CTCs by the AutoMACS device. LP-1 cell concentration increased to 92% for 1% samples but only 7% for 0.01% samples. Purity decreased with lower concentrations but a 700-fold tumor cell enrichment was achieved. The decline in yield was less pronounced (from 69 to 52%) (Figure 2A). For samples with lower tumor cell concentration (10^−5^ to 10^−7^), cell yield and purity could not be accurately examined. However, tumor detection in 10^−6^ LP-1 mixtures was possible only after enrichment (Supplementary Table S1). The CD138-based cell enrichment strategy can increase tumor cell concentration and increase sensitivity of downstream applications.

#### 3.1.2. Concentrating DNA by Vacuum Evaporation

Four independent DNA samples (two with AE-eluted DNA and two with DNAse FW-eluted DNA) were analyzed with albumin PCR. DNA concentrations were higher after vacuum evaporation in both AE-eluted and DNAse FW-eluted samples, with similar concentration factors (Table 1). There is no PCR-inhibition by EDTA salts after the 4.5-fold concentration of AE buffer through vacuum evaporation. Thus, patient DNA samples were safely concentrated from 200 µL of AE buffer (per manufacturer’s protocol) to 60 µL (3.3×).

A two-step approach consisting of tumor cell enrichment followed by vacuum evaporation to maximize DNA-input for downstream applications resulted in better sensitivity.

### 3.2. Patient Samples

#### 3.2.1. Patients with Active Disease

**Figure 3:**
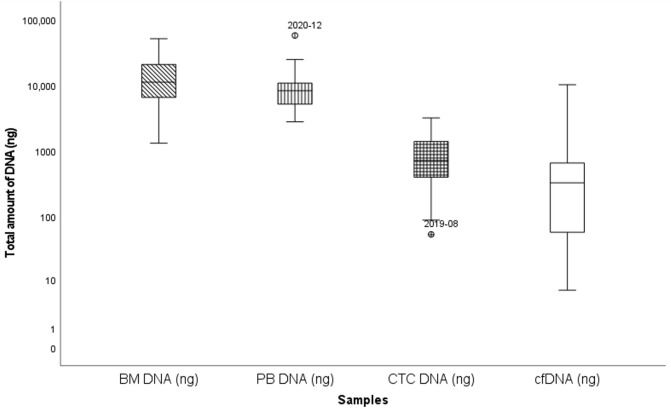
Box and whisker plot summarizing total DNA extracted from different sources, mean DNA concentration is indicated. *Y*-axis: total DNA (ng); *X*-axis: different DNA-sources. Outlier samples are individually mentioned.

Paired PBMNC, enriched CTC and cfDNA samples were available for 33 patients with detectable clonal sequences, and these were analyzed and compared with the BM sample. The median age was 71 (±8.4) years, there were 12 NDMM patients (36%), 3 smoldering myeloma patients and 18 RRMM patients (55%) (Table 2, Supplementary Table S2). The median amount of extracted cfDNA was 323 ng (range: 6.5 ng–10,140 ng) while this was 10,900 ng (range: 1300 ng–51,000 ng), 8325 ng (2760 ng–57,000 ng) and 659 ng (range: 52 ng–3160 ng) for BM, PBMNC and enriched CTC, respectively (Figure 3, Supplementary Table S3a). Overall, no clear differences were observed in the amounts of cfDNA, enriched CTC-DNA or PBMNC-DNA, although a trend toward higher cfDNA levels was noted in samples from patients with two or more relapses. Detection rates at diagnosis were comparable across NDMM and RRMM (67% in NDMM, 57% at first relapse and 100% in patients with two or more relapses; *p* = 0.138, Chi-square).

All NGS runs on BM samples had a standardized 50 ng DNA input and a median of 47,797 sequencing reads (range: 154–358,137). Median DNA input for NGS was 541 ng, 80 ng and 43 ng for PBMNC, enriched CTC and cfDNA, respectively. Sequencing depth was 108,507 (range: 781–453,892), 38,418 (range: 1040–353,912) and 4416 (range: 59–353,602), respectively. The myeloma sequence was detected in 29/33 PBMNC samples (88%) while all enriched CTC samples (33/33; 100%) were positive (*p* = 0.11; McNemar; Table 3). The median percentage of tumor cells was higher for enriched CTC (1.30%; 0.01–71.15%) compared to PBMNC samples (0.0085%; 0.00–4.74%. *p* < 0.0001; Wilcoxon signed-rank). For 29 paired PBMNC and enriched CTC samples, a median 37-fold enrichment was found (range: 4–2213). The myeloma specific clonal sequence was detected in 25/33 (76%) cfDNA samples, significantly less than in enriched CTC samples (*p* < 0.04; McNemar). Detection rates in three smoldering myeloma patients were not significantly different from patients with active disease. Only one cfDNA sample was negative for the clonal sequence.

In four PBMNC samples, the known clonal sequence could not be detected despite sufficient DNA input and sequencing depth (Supplementary Table S4). None of these patients had extramedullary (EM) disease. Conversely, both patients with proven non-adjacent EM disease had high clonal *IG* frequencies not found in the majority of patients without non-adjacent EM disease.

Clonal sequence detection in BM requires good sequencing depth and a relatively high proportion of reads (>5% of all reads) [40]. It is unknown if this is also true for liquid biopsies. Sequencing depth > 10,000× was reached in 33 PB (100%), 31 enriched CTC (94%) and 11 cfDNA (33%) samples. However, BM clonal sequences were not always the dominant sequence in liquid biopsies. Clonal sequences were present at a level of >5% in only 8 PBMNCs (24%), 21 enriched CTC (64%) and 10 cfDNA (30%) samples. Conversely, alternative “pseudoclonal” sequences (level > 5%, absent in BM) were found in 3/11 enriched CTC and/or cfDNA samples of myeloma patients with more than 10,000 reads. Moreover, abundant (>5%) sequences were found in the cfDNA of six healthy volunteers (without clonal disease), but only one sample had more than 10,000 reads.

**Table 3:** Comparison of detectable tumor sequences in DNA derived from different DNA sources.

	BM	PB	Enriched CTC	cfDNA
Clonal sequence detected	33 (100%)	29 (88%; *p* = 0.11)	33 (100%)	25 (76%; *p* < 0.04)
Clonal sequence not detected	0	4	0	8
Total	33	33	33	33

Comparison of positive samples from different sources. Significance (between brackets) is calculated for all samples compared to BM (gold standard). BM: bone marrow; PB: peripheral blood mononuclear cells; CTC: enriched circulating tumor cells; cfDNA: cell-free DNA.

#### 3.2.2. Patients in Remission

Thirty-seven patients in remission, with predetermined clonal sequences, were included. Patient characteristics are summarized in Table 4 (Supplementary Table S5).

**Figure 4:**
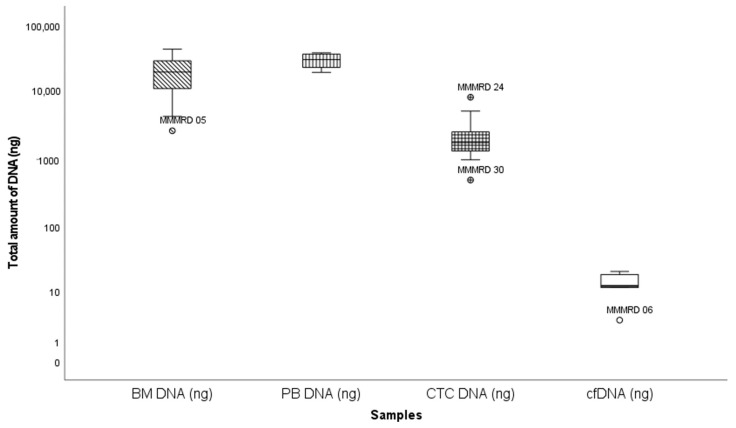
Box and whisker plot summarizing total DNA extracted from different sources. *Y*-axis: total DNA (ng); *X*-axis: different samples. Outlier samples are individually mentioned. Extreme values were excluded.

The median amount of DNA extracted from paired BM, PBMNC, enriched CTC and cfDNA samples was 20,200 ng (2660 ng–43,400 ng), 30,300 ng (19,600 ng–38,200 ng), 1739 ng (493 ng–8380 ng) and 15 ng (3 ng –165 ng), respectively (Figure 4). DNA amounts corrected for sample volume were significantly lower in cfDNA remission samples compared to active disease (36 vs. 1 ng; *p* < 0.001; Mann–Whitney), but not in enriched CTC samples (*p* = 0.30; Mann–Whitney). The comparable DNA amounts at remission and active disease reflect the use of higher blood volumes (and thus cell numbers) prior to enrichment. No significant difference was detected in the enriched CTC-DNA concentrations from NDMM patients and RRMM patients (*p* > 0.8; chi-square).

The median DNA input for BM MRD detection was 664 ng (range: 120 ng–1746 ng) per NGS reaction, and all reactions were run in duplicate. In comparison, the median DNA input was 963 ng (range: 600 ng–1386 ng), 131 ng (8.6 ng–600 ng) and 3.52 ng (0.55 ng–29.7 ng) for every PBMNC, enriched CTC and cfDNA reaction, respectively. A median sequencing depth of 241,610 (range: 8629–959,664), 566,121 (range: 113,064–1,230,931), 230,424 (range: 9649–1,515,513) and 29,233 (range: 2481–111,381) was reached for BM, PBMNC, enriched CTC and cfDNA samples, respectively. Residual disease was detected in 26/37 tested BM samples (70%). Twenty-four paired enriched CTC samples from BM MRD-positive patients were examined. Enriched CTC samples were positive for 12/24 BM MRD-positive patients. If BM MRD was below 10^−5^ (low MRD), no enriched CTC samples were positive (0/5). Only three/eight paired PBMNC samples from enriched CTC-positive patients showed residual disease. Our enrichment strategy increased CTCs up to 404-fold, improving sensitivity for clinical detection.

**Figure 5:**
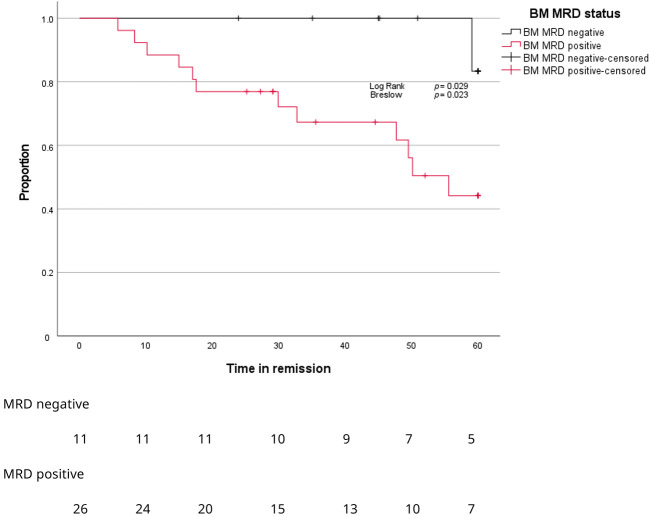
Kaplan–Meier curves for proportion of patients (*Y*-axis) progressing over time (*X*-axis) according to MRD stage in BM.

**Figure 6:**
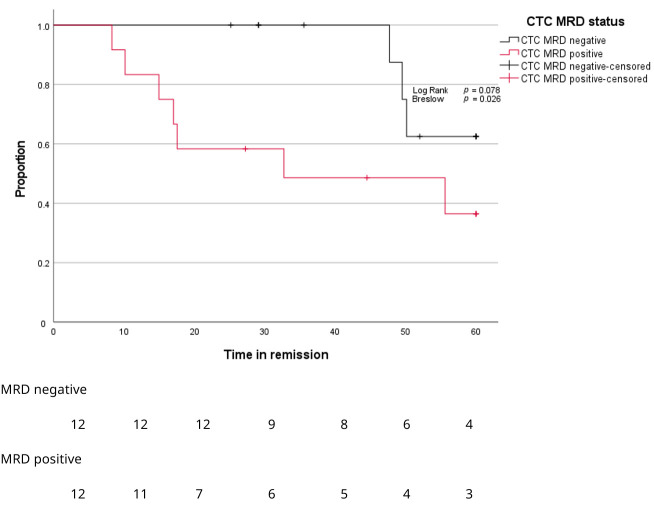
Kaplan–Meier curves for proportion of patients (*Y*-axis) progressing over time (*X*-axis) according to MRD stage in enriched CTC samples.

After a median follow-up of 45 months, BM MRD-negative patients showed significantly longer time to progression compared to BM MRD-positive patients (Figure 5, *p* = 0.029). Only BM MRD-positive patients were examined for the presence of circulating tumor cells. In this MRD-positive subgroup with a higher risk of relapse, enriched CTC-positivity associated with early relapse (log rank *p* = 0.078; Gehan–Breslow–Wilcoxon *p* = 0.026) (Figure 6). Five out of twelve enriched CTC-positive patients (42%) relapsed within 18 months. Conversely the first relapse in enriched CTC-negative patients occurred after almost 4 years (Figure 6). Differences in progression-free survival were less clear due to non-myeloma-related mortality, and two patients died from infections (COVID and pneumonia) during follow up. One BM MRD-negative patient relapsed after 59 months. An enriched CTC sample (collected simultaneously with the initial BM sample) was examined afterwards and did not detect any circulating tumor cells.

**Figure 7:**
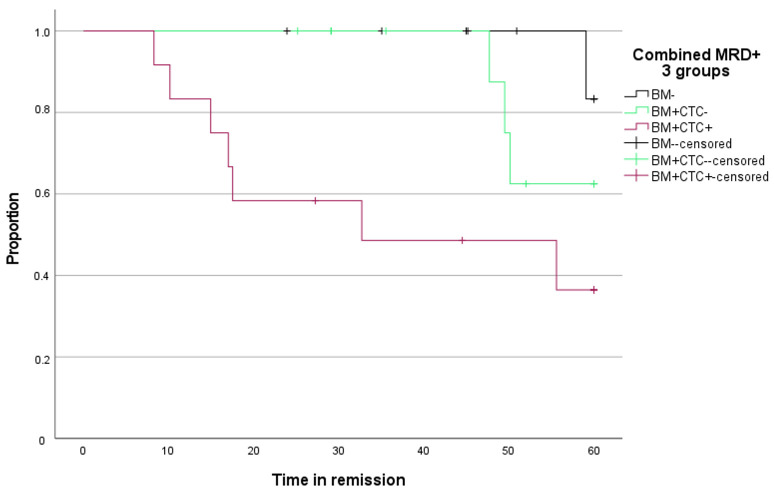
Kaplan–Meier curve for time to progression (*Y*-axis) over time (*X*-axis) according to MRD stage in BM and enriched CTC.

Time to progression for BM MRD-negative patients and BM MRD-positive, but enriched, CTC-negative patients was comparable. Time to progression for enriched CTC-positive patients was significantly shorter compared to BM MRD-negative patients (Figure 7, *p* = 0.007).

For three enriched CTC-negative patients who relapsed during follow-up, analysis of enriched CTC samples revealed the presence of tumor cell DNA up to 15 months prior to clinical or biochemical relapse.

## 4. Discussion

The detection of residual disease in BM samples of MM patients by Ig-targeted NGS is a reliable and sensitive prognostic tool that is increasingly being used to assess deep responses to therapy. However, this strategy has limited repeatability in clinical practice and requires an invasive sample collection, potentially making it less patient-friendly. Additionally, it is unable to detect extramedullary disease. Liquid biopsies could potentially address these issues, but it is currently unclear which DNA source from peripheral blood is most suitable for detecting MRD by NGS. To address this question, we compared in this study DNA derived from four different sources, using an NGS technique that we previously validated [38]. To the best of our knowledge, no other study has directly compared these four DNA sources for Ig-based tumor detection so far.

In the first instance, we used the LP-1 cell line to validate a dual-platform approach involving immunomagnetic CD138-targeted cell enrichment, followed by Ig-targeted NGS analysis. The first step of this method led to a significant (up to 700-fold) target cell enrichment, theoretically enabling the detection of myeloma clonotypes in peripheral blood (PB), with a sensitivity comparable to that of BM. Previous studies revealed, indeed, 40 to 300 times higher concentrations of myeloma cells in BM compared to peripheral blood [18,28,41]. In a series of blood samples from MM patients, we noted a median 37-fold enrichment of target cells, which was lower than the enrichment observed in the LP-1 cell line experiments, but in line with previously published findings on primary patient samples [41]. The initial concentrations of circulating tumor cells (CTCs) before enrichment, particularly in newly diagnosed and relapsed/refractory multiple myeloma cases, can impact the level of enrichment. Additionally, the inability to detect tumor cells before enrichment in some samples hampers the precise calculation of the enrichment factor for these cases. Lastly, the lower expression of CD138 on CTCs compared to human myeloma cell lines and BM myeloma cells may also lead to a lower enrichment level [42,43].

Following the extraction of DNA from suspensions with a lower cell count (such as enriched CTC), the DNA concentration in the final solution might be low and thus insufficient for sensitive tumor detection through NGS analysis. Vacuum evaporation has emerged as a valuable method for increasing DNA concentrations obtained from formalin-fixed, paraffin-embedded tissue samples, ensuring high quality and increased sensitivity for subsequent PCR or NGS analysis [44]. Our data suggest that utilizing this technique on DNA extracted from cell suspensions maintains DNA quality without inducing inhibitory effects.

Our NGS analyses successfully showed at least one Ig sequence (identified as clonal using the corresponding BM sample) in 88% of all tested PBMNC samples, while all enriched CTC samples were positive. These results are in line with various studies that have detected CTCs in the majority of peripheral blood samples from MM patients with active disease, using both molecular and flow cytometry-based methods [16,18,19,41,45]. Some of these studies also showed the added benefit of CTCs enrichment in achieving improved molecular tumor detection rates [18,41]. Although it has been recently suggested that cell-free DNA (cfDNA) could serve as a valuable matrix for myeloma detection, our findings suggest that utilizing this biomarker for Ig-targeted NGS results in lower tumor detection sensitivity, with a detection rate of 76%. This result aligns with findings from two other studies involving a combined total of 61 tested patients [18,32], but contrasts with three studies, including a combined total of 41 patients, with a 100% detection rate through Ig-sequencing-based cfDNA analysis [31,33,46]. Four out of five cited studies used the same extraction method as we did and reported similar cfDNA concentrations. Our observations also contrast with another recent study conducted by our group, where cfDNA was identified as the optimal blood-derived biomarker for mutation profiling in MM [47]. The primary reason for this discrepancy likely lies in the shorter fragment length of cfDNA compared to genomic DNA. The shorter length of DNA fragments required for targeted mutation analysis or whole genome sequencing, compared to the DNA length needed to facilitate Ig-amplicon generation for NGS analysis, can result in sub-optimal detection. For this reason, a Fr3 primer (targeting shorter fragments) was used when possible. Also, some IGK-amplicons are shorter in size and therefore more useful for cfDNA. This also suggests that the most suitable DNA biomarker (cell-free versus enriched CTC-derived) may vary depending on the specific target(s) used for tumor detection. Despite efforts to increase the DNA concentration through vacuum evaporation, cfDNA samples often contained lower DNA concentrations than PBMNC or enriched CTC samples. This DNA concentration affects sensitivity, thereby influencing detection rates.

Although enriched CTC-derived DNA, and to a lesser extent cfDNA, may effectively be suitable to detect myeloma Ig-sequences, previously identified using a matched BM sample at the time of diagnosis, both biomarkers are not optimal to determine clonality. The detected clonal sequences were not always dominant in liquid biopsy-derived DNA. The enrichment process for CTCs and the limited availability of amplifiable DNA in both enriched CTC and cfDNA contribute to the identification of “pseudoclonal sequences”. These relatively common sequences indicate imbalanced amplification and are not typically found when analyzing BM samples. Consequently, we believe liquid biopsies currently cannot substitute for BM biopsies in the primary identification of clonal immunoglobulin (Ig) sequences.

When using enriched CTC-DNA for Ig-targeted NGS in a relatively small cohort of MM patients in remission, myeloma cell detection was successful in half of the tested patients (50%) who also exhibited MRD positivity in matched BM samples. This exceeds the findings of earlier studies using PBMNC samples, where molecular detection rates (qASO-PCR) ranged from 25% to 37% [19,28]. In line with previous findings, our study confirmed that BM MRD-positivity was linked to a shorter time to progression and progression-free survival (PFS) [48]. The median time in remission if BM MRD-positive was 55 months; however, significant differences existed for enriched CTC-positive and enriched CTC-negative patients. BM MRD-positive, enriched CTC-negative patients had an excellent median overall survival of 76 months with the first relapse occurring after almost 4 years (47.7 months). However, BM MRD-positive, enriched CTC-positive patients had a worse prognosis with relapses within 18 months for 5/12 enriched CTC-positive patients. These results are obtained from a limited number of samples and should be confirmed in larger groups.

Therefore, the presence of CTCs (measured by Ig-targeted NGS) appears to be an early indicator of relapse. The absence of detectable enriched CTC-DNA corresponded with almost 4 years’ time to first progression in our group. Moreover, the few enriched CTC-negative patients that relapsed had detectable tumor sequences in the last sample prior to relapse (2–15 months before clinical relapse). Detection of CTCs may predict imminent relapse prior to detectable disease with conventional methods.

## 5. Conclusions

In summary, we have identified enriched circulating tumor cells (enriched CTC) as a useful circulating DNA source for liquid biopsy-based tumor detection of myeloma cells. Sensitivity for MRD of enriched CTC was found to be lower than BM-derived DNA. However, detection of enriched CTC-DNA in BM MRD-positive patients indicates impending relapses while BM MRD-positive patients relapsed much later when no enriched CTC–DNA was detected. These findings indicate that NGS using DNA from enriched CTC can identify a subset of patients who, despite having MRD detectable in the BM, have a more favorable prognosis. Enriched CTC-based NGS may therefore offer additional prognostic stratification beyond a conventional BM-based MRD assessment. Moreover, our data suggest that in patients with BM-positive but enriched CTC-negative NGS results in remission, less frequent bone marrow-based MRD monitoring may be sufficient, potentially reducing the burden of invasive procedures without compromising clinical follow-up. These findings necessitate further verification through the assessment of larger patient cohorts and longer follow-up periods. Nonetheless, we can assert that enriched CTC offer a promising biomarker for immunoglobulin-targeted NGS, facilitating a less invasive and more patient-centered approach for monitoring disease progression and assessing therapy responses in multiple myeloma.

## Figures and Tables

**Table 1:** Evaluation of evaporation efficiency and downstream PCR analysis.

	Starting Sample (ng/µL)	Evaporated Sample (ng/µL)	Concentration Factor
H_2_O eluted sample 1	1.36	6.76	4.97
H_2_O eluted sample 2	1.25	7.31	5.85
AE eluted sample 3	0.66	3.14	4.76
AE eluted sample 4	1.39	7.012	5.05

Comparison of DNA concentrations as measured by a quantitative albumin PCR in original samples, listed in column 2. Column 3 shows the DNA concentrations in ng/µL after evaporation. Observed concentration factors are indicated in column 4 and close to theoretical concentration factor (4.5×). AE: elution buffer. H_2_O: DNAse- and RNAse-free water.

**Table 2:** Active disease patient demographics and characteristics.

	Total Number
**Total Patients**	**33**
**Age (min-max) (in years)**	**71 (48–85)**
**Disease stage**	
SMM (%)	3 (9)
NDMM (%)	12 (36)
RRMM (%)	18 (55)
**Sex**	
Male (%)	15(45)
Female (%)	18 (55)
**IgG subtype**	
IgG (%)	23 (70)
IgA (%)	5 (15)
Light chain (%)	2 (6)
Other (%)	3 (9)
**IG-gene**	
IGH (%)	25 (76)
IGK (%)	29 (88)
IGH and IGK (%)	21 (64)
**Extramedullary disease**	
Yes (paraskeletal and true EM) (%)	6 (18)
Yes (true EM) (%)	2 (6)
No (%)	25 (76)
No data (%)	2 (6)

Characteristics of 33 patients with paired samples BM, PBMNCs, enriched CTC and cfDNA. NDMM: newly diagnosed multiple myeloma; RRMM: relapsed/refractory multiple myeloma; IGH: immunoglobulin heavy chain gene; IGK: immunoglobulin light chain kappa gene; EM: extramedullary.

**Table 4:** Remission patient demographics and characteristics.

	**Total Number (%)**
**Total** **patients**	37
**Age** **(min-max)** **(in years)**	65 (47–82)
**Disease stage**	
- NDMM (%)	26 (70)
- RRMM (%)	11 (30)
- 2nd line	8 (22)
- 3 or more lines	3 (8)
**Sex**	
- Male (%)	20 (54)
- Female (%)	17 (46)
**IgG subtype**	
- IgG (%)	24 (65)
- IgA (%)	4 (11)
- Light chain (%)	9 (24)
- Other (%)	0 (0)
**IG-gene**	
- IGH (%)	23 (62)
- IGK (%)	31 (84)
- IGH and IGK (%)	17 (46)
**Extramedullary disease**	
- Yes (continuous and true EM) (%)	0 (0)
- No (%)	37 (100)

Characteristics of 37 patients in remission with paired BM, PBMNC, enriched CTC and cfDNA samples. NDMM: newly diagnosed multiple myeloma; RRMM: relapsed/refractory multiple myeloma; IGH: immunoglobulin heavy chain gene; IGK: immunoglobulin light chain kappa gene; EM: extramedullary.

## Data Availability

The data presented in this study are available on request from the corresponding author due to European privacy regulation.

## Supplementary Materials

The following supporting information can be downloaded at: https://www.mdpi.com/article/10.3390/cancers17244008/s1, Table S1: comparison of LP-1 detection prior to and after enrichment; Table S2: clinical information of patients with active disease; Table S3a,b: median total DNA extracted from samples; Table S4: DNA input and number of reads in negative PBMNC samples; Table S5: clinical information of patients in remission.
